# Complicated Pocket Infection in Patients Undergoing Lead Extraction: Characteristics and Outcomes

**DOI:** 10.3390/jcm12134397

**Published:** 2023-06-29

**Authors:** Anat Milman, Anat Wieder-Finesod, Guy Zahavi, Amit Meitus, Saar Kariv, Yuval Shafir, Roy Beinart, Galia Rahav, Eyal Nof

**Affiliations:** 1 Leviev Heart Institute, The Chaim Sheba Medical Center, Tel Hashomer, Ramat Gan 5262000, Israeleyalnof.dr@gmail.com (E.N.); 2 Sackler School of Medicine, Tel Aviv University, Tel Aviv 6997801, Israel; anat.wieder@sheba.health.gov.il (A.W.-F.);; 3 The Infectious Diseases Unit, Sheba Medical Center, Tel-Hashomer, Ramat Gan 5262000, Israel; 4 Department of Anesthesiology and Intensive Care, The Chaim Sheba Medical Center, Tel Hashomer, Ramat Gan 5262000, Israel

**Keywords:** cardiac implantable electronic device, transvenous lead extraction, infection

## Abstract

Cardiac implantable electronic device (CIED) infection can present with pocket or systemic manifestations, both necessitating complete device removal and pathogen-directed antimicrobial therapy. Here, we aim to characterize those presenting with both pocket and systemic infection. A retrospective analysis of CIED extraction procedures included 300 patients divided into isolated pocket (n = 104, 34.7%), complicated pocket (n = 54, 18%), and systemic infection (n = 142, 47.3%) groups. The systemic and complicated pocket groups frequently presented with leukocytosis and fever > 37.8, as opposed to the isolated pocket group. *Staphylococcus aureus* was the most common pathogen in the systemic and complicated pocket groups (43.7% and 31.5%, respectively), while Coagulase-negative staphylococci (CONS) predominated (31.7%) in the isolated pocket group (10.6%, *p* < 0.001). No differences were observed in procedural success or complications rates. Kaplan–Meier survival analysis found that at three years of follow-up, the rate of all-cause mortality was significantly higher among patients with systemic infection compared to both pocket groups (*p* < 0.001), with the curves diverging at thirty days. In this study, we characterize a new entity of complicated pocket infection. Despite the systemic pattern of infection, their prognosis is similar to isolated pocket infection. We suggest that this special category be presented separately in future publications of CIED infections.

## 1. Introduction

Over the past decade, there has been a dramatic increase in the number of cardiac implantable electronic devices (CIED) [1,2]. As a result, an exponential rise in transvenous lead extraction (TLE) procedures has evolved. TLE has exceeded the increase in implantation rates [3,4,5,6,7].

Infection is a serious complication after CIED implantation [1], necessitating complete device removal and pathogen-directed antimicrobial drug therapy [2]. Infections from CIEDs are costly, associated with substantial in-hospital and long-term mortality [3]. Optimal management of CIED infection at initial presentation is critical to reduce infection-associated morbidity, mortality, hospital length of stay, and relapse [4,7].

CIED infections can have different presentations. Classically, patients are divided into pocket vs. systemic infection [8]. Pocket infection typically presents with inflammatory changes at the pocket site, including erythema, swelling, pain, warmth, drainage, purulence, erosion, and dehiscence. Pocket infection may involve intravascular or intracardiac portions of leads, and if this situation results in bacteremia, lead infection, or endocarditis, systemic symptoms are prominent as well. The majority of patients present within 12 months of device placement or revision [9]. The pathogenesis is seeding of the pathogen from the skin to the generator. Systemic infection, on the other hand, presents with a primary bloodstream infection (bacteremia, lead infection, or endocarditis), without signs of pocket infection. The suggested mechanism is hematogenous seeding of device leads or heart valves from a distant source of bacteremia. Diagnosis of systemic CIED infections can be challenging and is often delayed [10,11].

Staphylococcal species are responsible for 60–80% of CIED infections [10,11]. *Staphylococcus aureus* is a notably virulent bacterium accounting for 25% of CIED infections, which often result in acute onset of fever and rigors. Coagulase-negative staphylococcus is the most common cause of device pocket-related infection but is less virulent and has fewer systemic symptoms [12,13]. Gram-negative bacilli account for 6–10%, while other Gram-positive pathogens, fungi, and skin flora account for an even lesser percentage. The pathogenesis of the CIED infection influences the microbiology and the clinical outcome.

Both pocket infections that are complicated with systemic infection and systemic infections without pocket infection may result in systemic inflammatory response syndrome (SIRS) criteria (fever or hypothermia, tachycardia, tachypnea, and leukocytosis or leukopenia) and/or hypotension (systolic blood pressure < 90 mm Hg or a >40 mm Hg drop from baseline). The aim of this study is to characterize those with complicated pocket infections, since these patients are not well-characterized, and compare them to patients with systemic only and pocket only CIED infections.

## 2. Methods

A retrospective analysis of all consecutive CIED extraction procedures at the Sheba Medical Center from July 2010 to December 2018 was performed. Demographic, clinical, laboratory, imaging, and microbiologic data were extracted from each chart. Device removal and associated complications were documented.

### 2.1. TLE Procedure

All TLE procedures were performed with a cardiothoracic surgeon immediately available on site. Patients were under general anesthesia, with hemodynamic monitoring. A large-bore femoral venous access was inserted in all patients. The procedure was performed by qualified experienced operators. A stepwise approach was used in all patients, as previously described by our group [14]. The TLE procedure was terminated after complete removal of the leads, or when lead fragments could not be removed or in the event of a major complication.

Complications were divided into major complications (defined as those that threaten life, such as tamponade, required surgical intervention, or resulted in death). Complications that did not meet the major complication criteria were classified as minor complications.

Success or failure was defined by the radiological findings, not clinical. Patients were divided into three groups depending on the outcome of the extraction procedure:

1. ‘Complete success’ was classified as the removal of the entire lead system.
2. ‘Partial success’ was defined as when most of the lead was removed, leaving at most 4 cm of coil and/or insulation and/or lead tip.
3. ‘Failure’ was defined if more than ≥4 cm of the tip remained.

All extraction patient records were reviewed and procedures due to infection were identified. Inclusion criteria were all patients undergoing extraction due to CIED-related infections, either carrying a permanent pacemaker (PPM) or implantable cardioverter defibrillator (ICD). Out of these, only records with confirmed culture growth, either pocket, blood, and/or lead cultures, were included in the study population. Patient data were collected and analyzed in accordance with the Sheba Helsinki Committee authorization for this study.

Outcome data were collected from records of follow-up visits to our outpatient clinic or hospitalization records. Due to the large volume of patients referred from other medical centers solely for extraction, some patients were not included in the follow-up. Mortality data were extracted from an Israeli governmental registry; thus, mortality rates were accurate for all patients, regardless of clinical follow-up.

For the purpose of this analysis, patients were firstly divided into pocket and systemic CIED infection, and then further divided into three groups:

*Isolated pocket infection:* Infection limited to the CIED pocket, such as localized cellulitis, swelling, discharge, dehiscence, or pain, with or without signs of fever. These patients have negative blood cultures and no evidence of a lead/valve vegetation on transesophageal echocardiogram (TEE).

*Complicated pocket infection:* pocket infection with positive blood cultures consistent with CIED infection or lead/valve vegetation seen on echocardiography.

*Systemic infection:* bacteremia or vegetations without signs or symptoms of pocket infection, including CIED-associated native or prosthetic valve endocarditis (CIED-IE) with no signs of generator pocket infection.

The following pathogen groups were predefined for analysis: *Staphylococcus aureus* (SA), Coagulase-negative staphylococcus (CONS), *Streptococcus* spp., *Enterococcus* spp., *Pseudomonas aeruginosa*, Enterobacteriaceae (*Escherichia coli*, *Citrobacter koseri*, *Klebsiella* spp., and more), and skin flora bacteria (*Corynebacterium* spp., *Cutibacterium* (formerly *Propionibacterium*) *acnes*), Candida, and mixed bacteria.

### 2.2. Principles of Antimicrobial Therapy

The duration and type of antimicrobial therapy was based on the infection group (isolated pocket, complicated pocket, and systemic) and culture results with susceptibility testing. The main recommendations following CIED removal were, for isolated pocket infections: treatment of 10 to 14 days with IV or PO antimicrobials, with a longer duration for deep wounds and wounds that underwent extra debridement and surgical procedures. Complicated pocket infections were treated according to the infection characteristics: infections with positive blood cultures and/or valve vegetations were treated for 6 weeks. Infections with lead vegetations and negative blood cultures, and no involvement of other cardiac structures by echocardiography, were treated for 4 weeks with IV or PO antimicrobials (or oral switch after starting IV), depending on the specific pathogen. Systemic infections were treated for 6 weeks with IV therapy. Specific treatment regimens were as recommended by the guidelines [1,2,15].

### 2.3. Statistical Analysis

Continuous variables are shown as mean ± SD or as median (IQR), and categorical variables as n (%). Variables were compared with an ANOVA test, Kruskal–Wallis test, or a Pearson’s chi-squared test. The survival probability at specific time points was estimated with logistic regression. All predictors with a significant hazard ratio or odds ratio (*p*-value ≤ 0.05) in univariate analysis were included in multivariate prediction models. For variables included in multivariate models, missing data were imputed if at least 75% of the data were complete. Missing data about medical diagnoses and procedural complications were marked as ‘No’. Kaplan–Meier survival curves were compared using the log-rank test. Significant *p*-values were considered when *p* < 0.05. All statistical analyses were conducted with R version 3.6.1 from R Foundation for Statistical Computing (Vienna, Austria).

## 3. Results

### 3.1. Demographic, Clinical, and Device Data

#### 3.1.1. Patient Characteristics

A total of 300 patients were eligible for the study. Baseline characteristics are shown in Table 1. Out of those, 158 (52.7%) had a pocket infection (divided into 104 (65.8%) isolated pocket infections and 54 (34.2%) complicated) and 142 (47.3%) had a systemic infection which did not involve the pocket. In the entire cohort, most of the patients were males, with a mean age of 66.6 ± 15.5 years at the time of extraction. Their comorbidities and lab results are listed in Table 1. More than half of the extracted devices were pacemakers (59.7%), followed by cardiac resynchronization therapy defibrillators (CRTD) (24.7%), implantable cardiac defibrillators (ICD) (12.7%), and cardiac resynchronization therapy pacemakers (CRTP) (3%).

The only differences in comorbidities that were observed between the groups included a significantly higher prevalence of diabetes (54.2%) in the systemic infection group compared to both pocket infection groups (33.7% and 35.2%) (Table 1).

Prosthetic valves were found in 9.7% of the patients. In patients with a systemic infection, 7.7% had a biologic prosthetic valve and 2.8% had a mechanical valve. In both pocket infection groups, mechanical valves were more prevalent than biological valves (5.8% isolated and 9.3% complicated, and 1.9% isolated and 1.9% complicated, respectively).

No difference was observed in the type of device extracted between all three groups (Table 1).

#### 3.1.2. Infection Manifestation

All groups had a similar rate of history of prior infection (Table 2). The prevalence of temperature higher than 37.8 °C was significantly different between all 3 groups (71.8% in the systemic infection group vs. 42.6% in the complicated pocket infection group vs. 8.7% in the isolated pocket infection group; *p* < 0.001) (Table 2). Leukocytosis was also more prevalent in the systemic group and the complicated pocket group compared to the isolated pocket infection group (50% vs. 35.2% vs. 22.1%, respectively, *p* < 0.001). Duration of antibiotic treatment was significantly longer for both the systemic and complicated pocket infection groups compared to the isolated pocket group (Table 2).

Patients with a systemic infection were sicker, as demonstrated by their lab results: higher creatinine (1.6 ± 1.1 mg/dL, *p* = 0.001), lower hemoglobin (10.2 ± 1.6 g/dL, *p* < 0.001), and lower albumin (2.8 ± 0.7 g/dL, *p* < 0.001). As expected, the complicated pocket infection group’s lab results were worse than those in the isolated pocket infection group (Table 2).

Positive pocket cultures were found in majority of both pocket infection groups (83.7% in the isolated and 88.9% in the complicated pocket infection groups) (Table 2). Positive blood cultures were more common in the systemic group (83.8%) than the complicated pocket group (53.7%) (*p* < 0.001). Positive lead cultures were relatively rare in all groups (mean 28.3%, *p* = 0.501).

Lead or valve vegetations were found in 28 patients (51.9%) of the complicated pocket group and in 74 patients (52.1%) of the systemic group. Most vegetations were demonstrated by transesophageal echocardiography (TEE) and not by transthoracic echocardiography (TTE) (TEE—52.1% and 51.9% for systemic and complicated pocket infection groups vs. TTE—11.3% and 16.7%, respectively; *p* < 0.001) (Table 2).

#### 3.1.3. Infectious Pathogens

**Figure 1:**
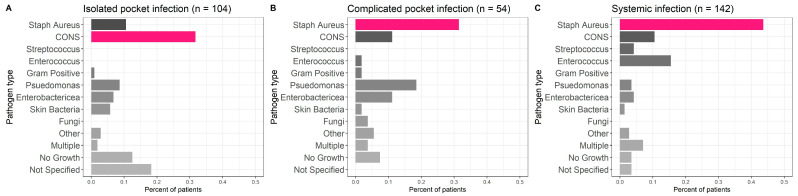
Infectious pathogens. *Staphylococcus aureus* was the most common pathogen responsible for CIED infection in the systemic (**C**) and complicated pocket infection (**B**) groups, while CONS was more frequent in the isolated pocket infection group (**A**).

SA was the most common pathogen responsible for CIED infection in the systemic and complicated pocket infection groups (43.7% and 31.5% vs. 10.6% in the isolated pocket group patients; *p* < 0.001), while CONS was more frequent in the isolated pocket infection group (31.7% vs. 11.1% in the complicated pocket group and 10.6% in the systemic group; *p* < 0.001, Figure 1). Gram-negative pathogens (especially pseudomonas) were more frequent in both pocket groups (15.4% in the isolated and 29.6% in the complicated pocket groups) compared to the systemic group (7.7%). CONS and skin pathogens were more frequent in the isolated pocket group (37.5% vs. 13% in the complicated pocket group and 12% in the systemic group) (Figure 1).

### 3.2. Outcomes

#### 3.2.1. Procedural Outcomes

A higher number of previous entries to the pocket were performed in the isolated and complicated pocket infection groups compared to the systemic infection group (2.4 ± 1.2 in the isolated and 2.2 ± 1.2 in the complicated pocket groups vs. 1.6 ± 0.9 in the systemic infection group, *p* < 0.001), with a temporal correlation from the last intervention to extraction (Table 3).

Complete removal of all leads (including tips) was achieved in 274/300 (91.3%) of the patients. In 18 patients (6%), partial removal was achieved, and in 6 patients (2%) the procedure was concluded as a failure (Table 3). Procedural success rates were achieved regardless of the etiology of extraction (*p* = 0.724) (Table 3). Complex tools were needed in most cases (69.7%), irrespective of the infection type (*p* = 0.078).

Major complications occurred in 7 (2.3%) patients and minor complications in 11 (3.7%) patients. Complication rates did not differ between groups (Table 3). Two patients died during the procedure and were excluded from further mortality analysis (one from each pocket infection group).

#### 3.2.2. Reinfection Outcomes

Reinfection at 30 days was almost exclusive to the systemic infection group (n = 9, 6.3%, vs. one patient from each pocket infection group (*p* = 0.108)) (Table 4), even though patients in the pocket infection groups were reimplanted with a permanent device more often than the systemic infection group (80.8% isolated and 90.7% complicated vs. 68.3% for the systemic group, *p* = 0.002) (Table 4).

#### 3.2.3. Mortality

**Figure 2:**
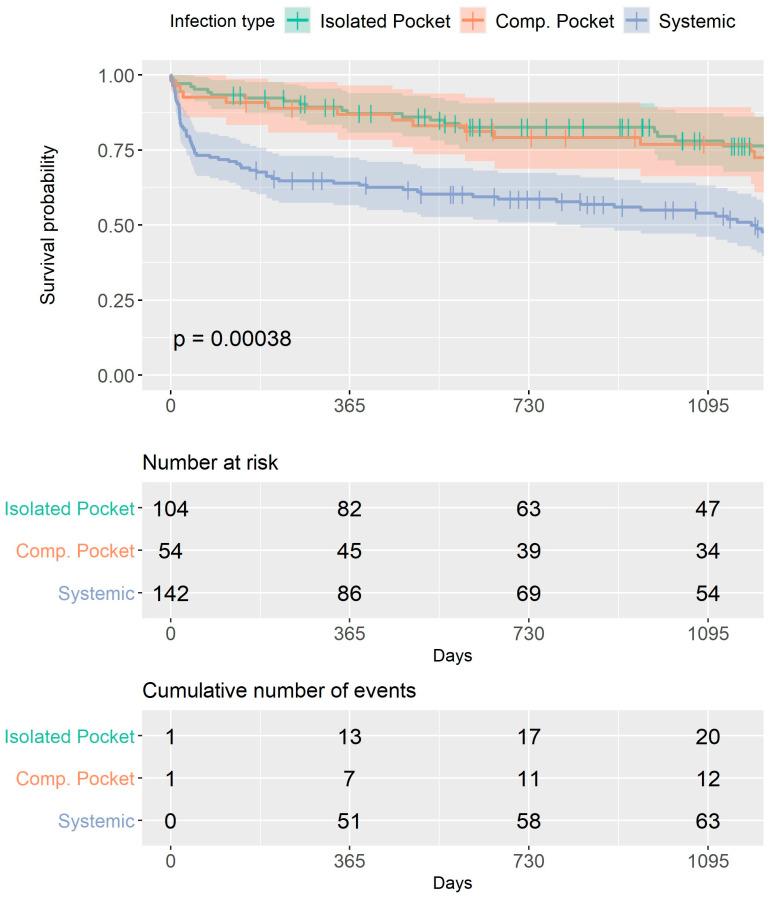
Overall survival for the entire study group. Kaplan–Meier survival analysis curves for each group. At 3 years of follow-up, the rate of all-cause mortality was significantly higher among patients with systemic infections compared to both pocket infection groups (*p* < 0.001), with the curves diverging at 30 days. All-cause mortality was similar between both pocket infection groups.

During 30 days after the procedure, 33 patients (11%) died, and at the 1-year follow-up, 71 (23.7%) patients had died. Kaplan–Meier survival analysis (Figure 2) showed that at 3 years of follow-up, the rate of all-cause mortality was significantly higher among patients with systemic infections compared to both pocket infection groups (*p* < 0.001), with the curves diverging at 30 days. All-cause mortality was similar between both pocket infection groups, regardless of if they had vegetations or positive cultures (Supplementary Table S1).

The univariate analysis showed that infection type, diabetes, lower hemoglobin, higher creatinine, leukocytosis, high temperature, lower albumin, and SA infection were all associated with short- and long-term mortality (Supplementary Table S2). Age, atrial fibrillation, heart failure, and vascular disease were found to predict long-term but not short-term mortality (Supplementary Table S3).

Multivariate analysis demonstrated that high creatinine and lower albumin were predictors of 30-day and 1-year mortality, while age and the presence of atrial fibrillation were predictors of 1-year mortality only (Table 5).

## 4. Discussion

Traditionally, patients rereferred for extraction were divided into pocket vs. systemic infection. This study characterized, for the first time, patients with complicated pocket infection, who differ from isolated pocket infection or systemic infection patients presenting for TLE. Most notably, these patients have special characteristics in the severity of their clinical presentation and microbiological pathogens; however, their outcome is significantly better than systemic infection and resembles those patients with isolated pocket infection.

CIED infections are usually categorized as either local pocket or endovascular, without differentiating the group of patients with positive BSI or vegetations secondary to generator pocket infection [16].

The present study distinguished between the two types of endovascular infections based on the involvement of the pocket site. This differentiation is important since the patients in each group have special characteristics and need different types of empiric therapy and treatment duration.

### 4.1. Patient Characteristics

The present study observed significant clinical differences between systemic and pocket infections. Diabetes seemed to be a risk factor for developing a systemic infection. In a previous meta-analysis, diabetes had an OR of 2.08 (1.62–2.67) for predicting CIED infection [17]; however, the meta-analysis did not distinguish the type of infection.

### 4.2. Infection Manifestation

Patients with a systemic infection had worse lab results, specifically those predicting a worse outcome, as described earlier by our group [18]. As expected, the lab results of the isolated pocket infection group were near normal, and those of the complicated pocket infection group were just in between the isolated and systemic groups.

Patients with complicated pocket infections had lower rates of fever and leukocytosis compared to those with systemic infections, perhaps suggesting that their diagnosis was earlier due to the local clinical signs and symptoms of pocket infection, while symptoms consistent with endocarditis resulted in a delayed diagnosis [11]. The complicated pocket infection group was treated as the systemic infection group, which was significantly longer than the isolated pocket infection group.

Although positive pocket cultures were obtained in majority of both pocket infection groups, a small percentage of the systemic group had positive pocket cultures as well, perhaps secondary to direct contamination of extracting infected leads through the pocket.

Blood cultures were positive in 83.8% and 53.7% of patients in the systemic only group and the complicated pocket group, respectively. This may also explain the better prognosis of the latter group and reflect the different pathogenesis of infection between the two groups.

Interestingly, the lead culture was positive in a similar percentage of all patients. TTE and TEE observed vegetations in a similar percentage of patients in the complicated pocket and systemic infection groups, probably related to SA, the major pathogen of these groups, as opposed to the higher CONS infection in the isolated pocket group (Figure 1). These results are in accordance with previous studies that compared systemic vs. pocket infections [19,20,21,22]. As was shown before [23], both pocket infection groups had a high percentage of Gram-negative bacteria in comparison to the systemic group.

### 4.3. Outcomes

Complete removal of all leads was achieved in most of our cohort (91%), with no difference in the tools used for the extraction procedure or complication rates between the groups. There were more entries to the pocket with a shorter time passing from the last intervention to infection manifestation for both pocket infection groups, signifying the casual relation between entering the pocket and its infection. This observation has been previously recognized and explained by repeated extractions and reimplantation, which could cause an inflammation and repeated bacterial colonization, leading to an infection [19].

In our study, 76.7% of patients were reimplanted with a permanent device within 46 ± 103 days, with 3.7% of the cohort suffering from reinfection during the first 30 days. These numbers are slightly higher than a previous study from the United States (1.3%) [24]. An intriguing finding of our study was the reinfection rate of 6.3% at 30 days for the systemic infection group, as opposed to that in each of the pocket infection groups, even though the latter groups were implanted more often with a permanent device after the procedure (*p* = 0.002). These findings deserve further attention in future studies in order to find which criteria are needed in choosing the patients for implantation of a CIED after undergoing a TLE. Other inflammatory markers, such as C-reactive protein and procalcitonin, could play a role in predicting the severity and type of the CIED infection. These parameters were not included in this analysis due to a lack of data.

### 4.4. Mortality

Mortality in our cohort was similar to that previously reported at one year (23.7%) [25,26]. An important observation is the worse outcome for the systemic group after three years of follow-up, with significantly higher mortality rates among patients with systemic infections compared to pocket infections, as was reported by the ELECTRA study [26], without a difference whether the pocket infection was complicated or not. Interestingly, our study showed, for the first time, a diversion in the Kaplan–Meier curves after 30 days post-TLE, as opposed to previous studies where no difference could be found in the mortality rates between groups after 12 months [27,28]. More significant is our finding of similar outcomes for each pocket infection group (complicated and isolated), implying that even though there are many similarities between the systemic infection and complicated pocket infection (hematogenous spread of infection resulting in infective endocarditis), these are different prognostic groups, which should be regarded as such. These findings are in contrast to a previous publication, where the type of CIED infection presentation was also a strong predictor for one-year mortality in patients with pocket infection who had positive blood cultures or vegetation on the leads (similar to our complicated pocket infection group), who had a one-year mortality that was higher than patients with pocket infection with negative blood cultures and no vegetation [25].

## 5. Conclusions

Patients with CIED infection due to a complicated pocket infection present with clinical characteristics of systemic infection that are milder than patients with endovascular infection alone. The most prevalent microbiological pathogen is SA; however, they have a high rate of Gram-negative bacteria as well. Despite the systemic pattern of the infection, their prognosis is similar to the isolated pocket infection group, and this might be due to the earlier diagnosis, wider range of causative pathogens, and lower rates of BSI. We suggest that this special category be presented separately in future publications of CIED infections. It is important to differentiate between all infection groups to provide the best treatment option and timing of CIED reimplantation.

## 6. Limitations

The main limitation of this study is that it was a retrospective analysis of a single center. Another limiting factor is that not all patients completed their follow-up, mainly those that were referred from other hospitals.

## Figures and Tables

**Table 1:** Baseline characteristics of the study group.

Infection Type		Overall	Pocket Infection		Systemic	*p* Value
			Isolated	Complicated		
**Number of patients**		300	104	54	142	
**Demographics**						
	Female	67 (22.3)	22 (21.2)	11 (20.4)	34 (23.9)	0.812
	Age (mean ± SD)	66.6 ± 15.5	66.6 ± 16.6	64.2 ± 18.8	67.5 ± 13.0	0.414
**Referral from other center**		205 (68.3)	71 (68.3)	35 (64.8)	99 (69.7)	0.805
**Comorbiditis**						
	Smoking	78 (26.0)	25 (24.0)	14 (25.9)	39 (27.5)	0.833
	Atrial fibrillation	110 (36.7)	40 (38.5)	13 (24.1)	57 (40.1)	0.102
	Hypertension	180 (60.0)	62 (59.6)	26 (48.1)	92 (64.8)	0.104
	Heart failure	135 (45.0)	44 (42.3)	26 (48.1)	65 (45.8)	0.758
	Stroke	42 (14.0)	14 (13.5)	4 (7.4)	24 (16.9)	0.227
	Vascular disease	168 (56.0)	58 (55.8)	27 (50.0)	83 (58.5)	0.566
	Malignancy	21 (7.0)	8 (7.7)	3 (5.6)	10 (7.0)	0.882
	Diabetes mellitus	131 (43.7)	35 (33.7)	19 (35.2)	77 (54.2)	0.002
	LVEF (%±SD)	40.5 ± 16.5	40.7 ± 15.4	39.4 ± 17.1	40.8 ± 17.2	0.878
**Prosthetic valve**						0.061
	Biological	14 (4.7)	2 (1.9)	1 (1.9)	11 (7.7)	
	Mechanical	15 (5.0)	6 (5.8)	5 (9.3)	4 (2.8)	
	No	271 (90.3)	96 (92.3)	48 (88.9)	127 (89.4)	
**Device type**						0.546
	CRT-D	74 (24.7)	29 (27.9)	13 (24.1)	32 (22.5)	
	CRT-P	9 (3.0)	4 (3.8)	3 (5.6)	2 (1.4)	
	ICD	38 (12.7)	14 (13.5)	8 (14.8)	16 (11.3)	
	PM	179 (59.7)	57 (54.8)	30 (55.6)	92 (64.8)	

Abbreviations: LVEF—left ventricular ejection fraction; CRT—resynchronization therapy; PM—pacemaker; ICD—implantable cardiac defibrillator.

**Table 2:** Infection manifestation.

Infection Type		Overall	Pocket		Systemic	*p* Value
			Isolated	Complicated		
**Number of patients**		300	104	54	142	
**Prior device infection**		44 (14.7)	19 (18.3)	9 (16.7)	16 (11.3)	0.278
**Temperature > 37.8 °C**						<0.001
	No	156 (52.0)	94 (90.4)	28 (51.9)	34 (23.9)	
	Yes	134 (44.7)	9 (8.7)	23 (42.6)	102 (71.8)	
	Unspecified	10 (3.3)	1 (1.0)	3 (5.6)	6 (4.2)	
**Lekuocytosis > 10K**		113 (37.7)	23 (22.1)	19 (35.2)	71 (50.0)	<0.001
**Duration of antibiotics (days)**		27.7 ± 19.8	16.5 ± 11.5	32.1 ± 24.6	34.1 ± 19.3	<0.001
**Lab results**						
	Creatinine (mg/dL)	1.4 ± 1.0	1.2 ± 0.7	1.3 ± 0.8	1.6 ± 1.1	0.001
	Hemoglobin (g/dL)	11.1 ± 1.9	12.0 ± 1.7	11.9 ± 1.5	10.2 ± 1.6	<0.001
	Albumin (g/dL)	3.2 ± 0.7	3.7 ± 0.5	3.5 ± 0.5	2.8 ± 0.7	<0.001
**Pocket Dehiscence**						<0.001
	Negative	179 (59.7)	25 (24.0)	15 (27.8)	139 (97.9)	
	Positive	117 (39.0)	78 (75.0)	39 (72.2)	0 (0.0)	
	Unspecified	4 (1.3)	1 (1.0)	0 (0.0)	3 (2.1)	
**Pocket Culture**						<0.001
	Negative	130 (43.3)	15 (14.4)	5 (9.3)	110 (77.5)	
	Positive	154 (51.3)	87 (83.7)	48 (88.9)	19 (13.4)	
	Not performed	10 (3.3)	0 (0.0)	0 (0.0)	10 (7.0)	
	Unspecified	6 (2.0)	2 (1.9)	1 (1.9)	3 (2.1)	
**Blood Culture**						<0.001
	Negative	149 (49.7)	102 (98.1)	24 (44.4)	23 (16.2)	
	Positive	148 (49.3)	0 (0.0)	29 (53.7)	119 (83.8)	
	Not performed	2 (0.7)	1 (1.0)	1 (1.9)	0 (0.0)	
	Unspecified	1 (0.3)	1 (1.0)	0 (0.0)	0 (0.0)	
**Lead Culture**						0.501
	Negative	205 (68.3)	67 (64.4)	39 (72.2)	99 (69.7)	
	Positive	85 (28.3)	31 (29.8)	14 (25.9)	40 (28.2)	
	Not performed	8 (2.7)	5 (4.8)	1 (1.9)	2 (1.4)	
	Unspecified	2 (0.7)	1 (1.0)	0 (0.0)	1 (0.7)	
**Transthoracic Echocardiography**						0.002
	No vegetation	166 (55.3)	61 (58.7)	34 (63.0)	71 (50.0)	
	Vegetation	25 (8.3)	0 (0.0)	9 (16.7)	16 (11.3)	
	Not performed	69 (23.0)	21 (20.2)	10 (18.5)	38 (26.8)	
	Unspecified	40 (13.3)	22 (21.2)	1 (1.9)	17 (12.0)	
**Transesophageal Echocardiography**						<0.001
	No vegetation	75 (25.0)	40 (38.5)	12 (22.2)	23 (16.2)	
	Vegetation	102 (34.0)	0 (0.0)	28 (51.9)	74 (52.1)	
	Not performed	29 (9.7)	22 (21.2)	3 (5.6)	4 (2.8)	
	Unspecified	94 (31.3)	42 (40.4)	11 (20.4)	41 (28.9)	

**Table 3:** Procedural details.

Infection Type	Overall	Pocket		Systemic	*p* Value
		Isolated	Complicated		
**Number of patients**	300	104	54	142	
**First device to extraction (days)**	2691.7 ± 2187.3	2966.6 ± 2417.3	2814.0 ± 2003.0	2442.9 ± 2058.2	0.163
**Current device to extraction (days)**	1394.7 ± 1485.7	1170.1 ± 1304.9	1293.9 ± 1527.0	1600.9 ± 1576.6	0.076
**Last intervention to extraction (days)**	907.3 ± 975.1	705.5 ± 842.1	521.2 ± 684.3	1183.8 ± 1068.3	<0.001
**Entries to pocket**	2.0 (1.1)	2.4 (1.2)	2.2 (1.2)	1.6 (0.9)	<0.001
**Extraction type**					0.078
**Simple**	88 (29.3)	31 (29.8)	9 (16.7)	48 (33.8)	
**Complex**	209 (69.7)	73 (70.2)	43 (79.6)	93 (65.5)	
**Unspecified**	3 (1.0)	0 (0.0)	2 (3.7)	1 (0.7)	
**Number of leads extracted**	2.2 (0.9)	2.3 (0.9)	2.4 (1.0)	2.2 (0.8)	0.314
**Extraction success**					0.724
**Full**	274 (91.3)	94 (90.4)	49 (90.7)	131 (92.3)	
**Partial**	18 (6.0)	8 (7.7)	2 (3.7)	8 (5.6)	
**Failure**	6 (2.0)	2 (1.9)	2 (3.7)	2 (1.4)	
**Unspecified**	2 (0.7)	0 (0.0)	1 (1.9)	1 (0.7)	
**Minor complications**	11 (3.7)	4 (3.8)	2 (3.7)	5 (3.5)	0.991
**Major complications**	7 (2.3)	3 (2.9)	2 (3.7)	2 (1.4)	0.572
**Temporary reimplant**	77 (25.7)	27 (26.0)	19 (35.2)	31 (21.8)	0.160
**Intra-procedural death**	2 (0.7)	1 (1.0)	1 (1.9)	0 (0.0)	0.327

**Table 4:** Reinfection outcomes.

Infection Type	Overall	Pocket		Systemic	*p* Value
		Isolated	Complicated		
**Number of patients**	300	104	54	142	
**Permanent reimplant**	230 (76.7)	84 (80.8)	49 (90.7)	97 (68.3)	0.002
**Time to reimplant (days)**	46 ± 103	53 ± 111	55 ± 78	34 ± 106	0.557
**Infection within 30 days**					0.108
**No**	134 (44.7)	53 (51.0)	25 (46.3)	56 (39.4)	
**Yes**	11 (3.7)	1 (1.0)	1 (1.9)	9 (6.3)	
**Unspecified**	155 (51.7)	50 (48.1)	28 (51.9)	77 (54.2)	

**Table 5:** Multivariate models for the prediction of mortality.

At 30 days				
**Mortality at 30 days**	**Odds ratio**	**Lower CI 95%**	**Upper CI 95%**	***p* value**
**Diabetes mellitus**	1.14	0.5	2.61	0.748
**Creatinine (mg/dL)**	1.43	1.03	1.99	0.034
**Albumin (g/dL)**	0.44	0.2	0.96	0.038
**Hemoglobin (g/dL)**	1.01	0.75	1.35	0.973
**Lekuocytosis > 10K**	1.43	0.63	3.25	0.392
**Temperature > 37.8**	0.79	0.29	2.17	0.654
**Staph aureus**	1.46	0.62	3.39	0.384
**Isolated pocket infection**	0.54	0.1	2.87	0.471
**Systemic infection**	1.43	0.42	4.89	0.557
At 1 year				
**Mortality at 1 year**	**Odds ratio**	**Lower CI 95%**	**Upper CI 95%**	***p* value**
**Age**	1.04	1	1.07	0.017
**Atrial fibrillation**	2.35	1.22	4.55	0.01
**Heart failure**	1.36	0.68	2.73	0.386
**Vascular disease**	1.11	0.53	2.29	0.785
**Diabetes mellitus**	1.04	0.52	2.09	0.905
**Creatinine (mg/dL)**	1.69	1.21	2.35	0.001
**Albumin (g/dL)**	0.33	0.17	0.64	< 0.001
**Hemoglobin (g/dL)**	1.12	0.88	1.43	0.339
**Lekuocytosis > 10K**	1.2	0.61	2.36	0.597
**Temperature > 37.8**	0.52	0.22	1.23	0.13
**Staph aureus**	1.34	0.64	2.8	0.433
**Isolated pocket infection**	1.06	0.32	3.48	0.925
**Systemic infection**	2.24	0.8	6.23	0.113

## Data Availability

The data presented in this study are available upon request from the corresponding author.

## Supplementary Materials

The following supporting information can be downloaded at: https://www.mdpi.com/article/10.3390/jcm12134397/s1, Table S1. The different mortality rates between groups at different time points. Table S2: Univariate analysis of 30-day mortality. Table S3. Univariate analysis of one-year mortality.

## References

[1] Brignole M., Auricchio A., Baron-Esquivias G., Bordachar P., Boriani G., Breithardt O.A., Cleland J.G.F., Deharo J.-C., Delgado V., Elliott P.M. (2013). 2013 ESC guidelines on cardiac pacing and cardiac resynchronization therapy: The task force on cardiac pacing and resynchronization therapy of the European Society of Cardiology (ESC). Developed in collaboration with the European Heart Rhythm Association (EHRA). Europace.

[2] Epstein A.E., Dimarco J.P., Ellenbogen K.A., Estes N.A., Freedman R.A., Gettes L.S., American College of Cardiology/American Heart Association Task Force on Practice, American Association for Thoracic Surgery, Society of Thoracic Surgeons (2008). ACC/AHA/HRS 2008 guidelines for Device-Based Therapy of Cardiac Rhythm Abnormalities: Executive summary. Heart Rhythm.

[3] Wilkoff B.L., Love C.J., Byrd C.L., Bongiorni M.G., Carrillo R.G., Crossley G.H., Epstein L.M., Friedman R.A., Ken-nergren C.E., Mitkowski P. (2009). Transvenous lead extraction: Heart Rhythm Society expert consensus on facilities, training, indications, and patient man-agement: This document was endorsed by the American Heart Association (AHA). Heart Rhythm.

[4] Baddour L.M., Epstein A.E., Erickson C.C., Knight B.P., Levison M.E., Lockhart P.B., Masoudi F.A., Okum E.J., Wilson W.R., Beerman L.B. (2010). Update on cardiovascular implantable electronic device infections and their management: A scientific statement from the American Heart Association. Circulation.

[5] Maytin M., Epstein L.M. (2011). The challenges of transvenous lead extraction. Heart.

[6] Deharo J.C., Bongiorni M.G., Rozkovec A., Bracke F., Defaye P., Fernandez-Lozano I., Golzio P.G., Hansky B., Kennergren C., Manolis A. (2012). Pathways for training and accreditation for transvenous lead extraction: A European Heart Rhythm Association position paper. Europace.

[7] Sohal M., Williams S.E., Arujuna A., Chen Z., Bostock J., Gill J.S., Rinaldi C.A. (2013). The current practice and perception of cardiac implantable electronic device transvenous lead extraction in the UK. Europace.

[8] Blomström-Lundqvist C., Traykov V., Erba P.A., Burri H., Nielsen J.C., Bongiorni M.G., Poole J., Boriani G., Costa R., Deharo J.C. (2020). European Heart Rhythm Association (EHRA) international consensus document on how to prevent, diagnose, and treat cardiac implantable electronic device infections-endorsed by the Heart Rhythm Society (HRS), the Asia Pacific Heart Rhythm Society (APHRS), the Latin American Heart Rhythm Society (LAHRS), International Society for Cardiovascular Infectious Diseases (ISCVID), and the European Society of Clinical Microbiology and Infectious Diseases (ESCMID) in collaboration with the European Association for Cardio-Thoracic Surgery (EACTS). Eur. Heart J..

[9] Kleemann T., Becker T., Strauss M., Dyck N., Weisse U., Saggau W., Burkhardt U., Seidl K. (2010). Prevalence of bacterial colonization of generator pockets in implantable cardioverter defibrillator patients without signs of infection undergoing generator replacement or lead revision. Europace.

[10] Chambers S.T. (2005). Diagnosis and management of staphylococcal infections of pacemakers and cardiac defibrillators. Intern. Med. J..

[11] Klug D., Lacroix D., Savoye C., Goullard L., Grandmougin D., Hennequin J.L., Kacet S., Lekieffre J. (1997). Systemic infection related to endocarditis on pacemaker leads: Clinical presentation and management. Circulation.

[12] Hussein A.A., Baghdy Y., Wazni O.M., Brunner M.P., Kabbach G., Shao M., Gordon S., Saliba W.I., Wilkoff B.L., Tarakji K.G. (2016). Microbiology of cardiac implantable electronic device infections. JACC Clin. Electrophysiol..

[13] Bongiorni M.G., Tascini C., Tagliaferri E., Di Cori A., Soldati E., Leonildi A., Zucchelli G., Ciullo I., Menichetti F. (2012). Microbiology of cardiac implantable electronic device infections. Europace.

[14] Younis A., Glikson M., Meitus A., Arwas N., Natanzon S.S., Lotan D., Luria D., Beinart R., Nof E. (2019). Transvenous lead extraction with laser reduces need for femoral approach during the procedure. PLoS ONE.

[15] Sandoe J.A., Barlow G., Chambers J.B., Gammage M., Guleri A., Howard P., Olson E., Perry J.D., Prendergast B.D., Spry M.J. (2015). Guidelines for the diagnosis, prevention and management of implantable cardiac electronic device infection. Report of a joint Working Party project on behalf of the British Society for Antimicrobial Chemotherapy (BSAC, host organization), British Heart Rhythm Society (BHRS), British Cardiovascular Society (BCS), British Heart Valve Society (BHVS) and British Society for Echocardiography (BSE). J. Antimicrob. Chemother..

[16] Tarakji K.G., Chan E.J., Cantillon D.J., Doonan A.L., Hu T., Schmitt S., Fraser T.G., Kim A., Gordon S.M., Wilkoff B.L. (2010). Cardiac implantable electronic device infections: Presentation, management, and patient outcomes. Heart Rhythm.

[17] Polyzos K.A., Konstantelias A.A., Falagas M.E. (2015). Risk factors for cardiac implantable electronic device infection: A systematic review and meta-analysis. Europace.

[18] Milman A., Zahavi G., Meitus A., Kariv S., Shafir Y., Glikson M., Luria D., Beinart R., Nof E. (2020). Predictors of short-term mortality in patients undergoing a successful uncomplicated extraction procedure. J. Cardiovasc. Electrophysiol..

[19] Hörnsten J., Axelsson L., Westling K. (2021). Cardiac Implantable Electronic Device Infections; Long-Term Outcome after Extraction and Antibiotic Treatment. Infect. Dis. Rep..

[20] Uslan D.Z., Sohail M.R., St Sauver J.L., Friedman P.A., Hayes D.L., Stoner S.M., Wilson W.R., Steckelberg J.M., Baddour L.M. (2007). Permanent pacemaker and implantable cardioverter defibrillator infection: A population-based study. Arch. Intern. Med..

[21] Fukunaga M., Goya M., Nagashima M., Hiroshima K., Yamada T., An Y., Hayashi K., Makihara Y., Ohe M., Ichihashi K. (2017). Identification of causative organism in cardiac implantable electronic device infections. J. Cardiol..

[22] Carrasco F., Anguita M., Ruiz M., Castillo J.C., Delgado M., Mesa D., Romo E., Pan M., De Lezo J.S. (2016). Clinical features and changes in epidemiology of infective endocarditis on pacemaker devices over a 27-year period (1987–2013). Europace.

[23] Esquer Garrigos Z., George M.P., Vijayvargiya P., Tan E.M., Farid S., Abu Saleh O.M., Friedman P.A., Steckelberg J.M., DeSimone D.C., Wilson W.R. (2019). Clinical Presentation, Management, and Outcomes of Cardiovascular Implantable Electronic Device Infections Due to Gram-Negative Versus Gram-Positive Bacteria. Mayo Clin. Proc..

[24] Boyle T.A., Uslan D.Z., Prutkin J.M., Greenspon A.J., Baddour L.M., Danik S.B., Tolosana J.M., Le K., Miro J.M., Peacock J. (2017). Reimplantation and Repeat Infection After Cardiac-Implantable Electronic Device Infections: Experience from the MEDIC (Multicenter Electrophysiologic Device Infection Cohort) Database. Circ. Arrhythm. Electrophysiol..

[25] Tarakji K.G., Wazni O.M., Harb S., Hsu A., Saliba W., Wilkoff B.L. (2014). Risk factors for 1-year mortality among patients with cardiac implantable electronic device infection undergoing transvenous lead extraction: The impact of the infection type and the presence of vegetation on survival. Europace.

[26] Bongiorni M.G., Kennergren C., Butter C., Deharo J.C., Kutarski A., Rinaldi C.A., Romano S.L., Maggioni A.P., Andarala M., Auricchio A. (2017). The European Lead Extraction ConTRolled (ELECTRa) study: A European Heart Rhythm Association (EHRA) Registry of Transvenous Lead Extraction Outcomes. Eur. Heart J..

[27] Nishii N., Morimoto Y., Miyoshi A., Tsukuda S., Miyamoto M., Kawada S., Nakagawa K., Watanabe A., Nakamura K., Morita H. (2019). Prognosis after lead extraction in patients with cardiac implantable electronic devices infection: Comparison of lead-related infective endocarditis with pocket infection in a Japanese single-center experience. J. Arrhythm..

[28] Ihlemann N., Møller-Hansen M., Salado-Rasmussen K., Videbæk R., Moser C., Iversen K., Bundgaard H. (2016). CIED infection with either pocket or systemic infection presentation—Complete device removal and long-term antibiotic treatment; long-term outcome. Scand. Cardiovasc. J..

