# Supplementary material for: Complicated Pocket Infection in Patients Undergoing Lead Extraction: Characteristics and Outcomes
Source: J Clin Med. 2023 Jun 29;12(13):4397. doi: 10.3390/jcm12134397 (PMC10342592; doi:10.3390/jcm12134397)
Supplement: Supplementary file 1 [file jcm-12-04397-s001.zip › jcm-2428133-supplementary.pdf]

Supplemental Materials

Supplemental Table S1. The different mortality rates between groups at different time points.

	Overall	Isolated	Complicated	Systemic	P value
Number of patients	300	104	42	154	
Mortality at 30 days	33 (11.0)	3 (2.9)	4 (9.5)	26 (16.9)	0.002
Mortality at 90 days	50 (16.7)	7 (6.7)	4 (9.5)	39 (25.3)	<0.001
Mortality at 1 year					0.001
No	213 (71.0)	82 (78.8)	34 (81.0)	97 (63.0)	
Yes	71 (23.7)	13 (12.5)	6 (14.3)	52 (33.8)	
Unspecified	16 (5.3)	9 (8.7)	2 (4.8)	5 (3.2)	
Mortality at 3 years					<0.001
No	135 (45.0)	47 (45.2)	25 (59.5)	63 (40.9)	
Yes	95 (31.7)	20 (19.2)	9 (21.4)	66 (42.9)	
Unspecified	70 (23.3)	37 (35.6)	8 (19.0)	25 (16.2)	
Mortality at 5 years					0.001
No	57 (19.0)	22 (21.2)	10 (23.8)	25 (16.2)	
Yes	122 (40.7)	27 (26.0)	15 (35.7)	80 (51.9)	
Unspecified	121 (40.3)	55 (52.9)	17 (40.5)	49 (31.8)	

Supplemental Table S2. Univariate analysis of 30days mortality.

Mortality at 30 days	All	Alive	Dead	p_value	Odds ratio	Lower CI 95%	Upper CI 95%
Number of patients	300	267	33				
Female	67 (22)	58 (22)	9 (27)	0.479	1.35	0.6	3.07
Age	67 (15)	66 (16)	70 (11)	0.216	1.02	0.99	1.04
Smoking	78 (26)	67 (25)	11 (33)	0.32	1.49	0.69	3.24
Atrial fibrillation	110 (37)	93 (35)	17 (52)	0.065	1.99	0.96	4.12
Hypertension	180 (60)	158 (59)	22 (67)	0.403	1.38	0.64	2.96
Heart failure	135 (45)	117 (44)	18 (55)	0.244	1.54	0.74	3.18
Stroke	42 (14)	36 (13)	6 (18)	0.478	1.43	0.55	3.69
Vascular disease	168 (56)	147 (55)	21 (64)	0.345	1.43	0.68	3.02
Malignancy	21 (7)	19 (7)	2 (6)	0.819	0.84	0.19	3.79
Prosthetic valve	29 (10)	25 (9)	4 (12)	0.624	1.34	0.43	4.11
Diabetes mellitus	131 (44)	111 (42)	20 (61)	0.038	2.16	1.03	4.53
ICD implant (not pacemaker)	112 (37)	102 (38)	10 (30)	0.37	0.7	0.32	1.54
History of device related infection	44 (15)	42 (16)	2 (6)	0.103	0.35	0.08	1.5
Number of leads extracted	2.2 (0.9)	2.2 (0.9)	2.3 (1)	0.57	1.12	0.75	1.68
First device to extraction (days)	2692 (2187)	2722 (2224)	2448 (1877)	0.487	0.9999	0.9998	1.0001
Extraction type	209 (70)	187 (71)	22 (67)	0.625	0.82	0.38	1.78
Creatinine (mg/dl)	1.4 (1)	1.3 (0.9)	2.2 (1.4)	< 0.001	1.93	1.42	2.63
Albumin (g/dl)	3.2 (0.7)	3.3 (0.7)	2.6 (0.5)	< 0.001	0.23	0.12	0.43
Hemoglobin (g/dl)	11.1 (1.9)	11.2 (1.8)	10 (1.7)	< 0.001	0.66	0.52	0.84
Lekuocytosis > 10K	113 (38)	94 (35)	19 (58)	0.014	2.5	1.2	5.21
Temperature > 37.8	134 (46)	112 (43)	22 (69)	0.006	2.87	1.31	6.3
Staph aureus	94 (34)	76 (31)	18 (60)	0.002	3.34	1.53	7.27
Streptococcus	8 (3)	7 (3)	1 (3)	0.857	1.22	0.15	10.28
Enterococcus	25 (9)	22 (9)	3 (10)	0.805	1.18	0.33	4.19
Pseudomonas	28 (10)	25 (10)	3 (10)	0.973	1.02	0.29	3.61
Simple pocket infection	104 (35)	101 (38)	3 (9)	< 0.001	0.16	0.05	0.55
Complicated pocket infection	54 (18)	50 (19)	4 (12)	0.33	0.6	0.2	1.78
Systemic infection	142 (47)	116 (43)	26 (79)	< 0.001	4.83	2.03	11.53

Supplemental Table S3. Univariate analysis of 1 year mortality.

Mortality at 1 year	All	Alive	Dead	p_value	Odds ratio	Lower CI 95%	Upper CI 95%
Number of patients	284	213	71				
Female	62 (22)	44 (21)	18 (25)	0.412	1.3	0.7	2.45
Age	66 (15)	65 (16)	72 (12)	< 0.001	1.04	1.02	1.06
Smoking	77 (27)	58 (27)	19 (27)	0.939	0.98	0.53	1.79
Atrial fibrillation	100 (35)	62 (29)	38 (54)	< 0.001	2.8	1.61	4.87
Hypertension	173 (61)	125 (59)	48 (68)	0.179	1.47	0.83	2.59
Heart failure	127 (45)	87 (41)	40 (56)	0.023	1.87	1.09	3.22
Stroke	40 (14)	27 (13)	13 (18)	0.249	1.54	0.75	3.19
Vascular disease	162 (57)	114 (54)	48 (68)	0.036	1.81	1.03	3.19
Malignancy	20 (7)	14 (7)	6 (8)	0.599	1.31	0.48	3.55
Prosthetic valve	29 (10)	19 (9)	10 (14)	0.228	1.67	0.74	3.79
Diabetes mellitus	124 (44)	84 (39)	40 (56)	0.013	1.98	1.15	3.41
ICD implant (not pacemaker)	106 (37)	76 (36)	30 (42)	0.324	1.32	0.76	2.28
History of device related infection	40 (14)	34 (16)	6 (8)	0.099	0.49	0.2	1.21
Number of leads extracted	2.3 (0.9)	2.2 (0.9)	2.3 (0.9)	0.49	1.11	0.82	1.5
First device to extraction (days)	2677 (2163)	2749 (2289)	2464 (1731)	0.328	0.9999	0.9998	1.0001
Extraction type	203 (72)	155 (74)	48 (68)	0.318	0.74	0.41	1.33
Creatinine (mg/dl)	1.4 (1)	1.2 (0.7)	2.1 (1.3)	< 0.001	2.58	1.8	3.68
Albumin (g/dl)	3.2 (0.7)	3.4 (0.6)	2.7 (0.6)	< 0.001	0.19	0.11	0.32
Hemoglobin (g/dl)	11 (1.9)	11.3 (1.8)	10.2 (1.7)	< 0.001	0.69	0.58	0.83
Lekuocytosis > 10K	111 (39)	75 (35)	36 (51)	0.022	1.89	1.1	3.26
Temperature > 37.8	131 (48)	92 (44)	39 (59)	0.032	1.84	1.05	3.22
Staph aureus	91 (35)	59 (30)	32 (50)	0.004	2.36	1.32	4.19
Streptococcus	7 (3)	4 (2)	3 (5)	0.274	2.4	0.52	11.03
Enterococcus	24 (9)	16 (8)	8 (12)	0.293	1.64	0.67	4.02
Pseudomonas	26 (10)	21 (10)	5 (8)	0.519	0.72	0.26	2
Simple pocket infection	95 (33)	82 (38)	13 (18)	0.001	0.36	0.18	0.69
Complicated pocket infection	52 (18)	45 (21)	7 (10)	0.025	0.41	0.18	0.95
Systemic infection	137 (48)	86 (40)	51 (72)	< 0.001	3.77	2.1	6.76
